# The impact of the Calman–Hine report on the processes and outcomes of care for Yorkshire's colorectal cancer patients

**DOI:** 10.1038/sj.bjc.6603372

**Published:** 2006-10-17

**Authors:** E Morris, R A Haward, M S Gilthorpe, C Craigs, D Forman

**Affiliations:** 1 Cancer Epidemiology Group, Centre for Epidemiology and Biostatistics, The University of Leeds, Arthington House, Cookridge Hospital, Leeds LS16 6QB, UK; 2 Northern & Yorkshire Cancer Registry and Information Service, Arthington House, Cookridge Hospital, Leeds LS16 6QB, UK; 3 Biostatistics Unit, Centre for Epidemiology and Biostatistics, The University of Leeds, 30-32 Hyde Terrace, Leeds LS2 9LN, UK

**Keywords:** colorectal cancer, Calman-Hine, specialisation, multidisciplinary teams

## Abstract

The 1995 Calman–Hine plan outlined radical reform of the UK's cancer services with the aim of improving outcomes and reducing inequalities in NHS cancer care. Its main recommendation was to concentrate care into the hands of site-specialist, multi-disciplinary teams. This study aimed to determine if the implementation of Calman–Hine cancer teams was associated with improved processes and outcomes of care for colorectal cancer patients. The design included longitudinal survey of 13 colorectal cancer teams in Yorkshire and retrospective study of population-based data collected by the Northern and Yorkshire Cancer Registry and Information Service. The population was all colorectal cancer patients diagnosed and treated in Yorkshire between 1995 and 2000. The main outcome measures were: variations in the use of anterior resection and preoperative radiotherapy in rectal cancer, chemotherapy in Dukes stage C and D patients, and five-year survival. Using multilevel models, these outcomes were assessed in relation to measures of the extent of Calman–Hine implementation throughout the study period, namely: (i) each team's degree of adherence to the Manual of Cancer Service Standards (which outlines the specification of the ‘ideal’ colorectal cancer team) and (ii) the extent of site specialisation of each team's surgeons. Variation was observed in the extent to which the colorectal cancer teams in Yorkshire had conformed to the Calman–Hine recommendations. An increase in surgical site specialisation was associated with increased use of preoperative radiotherapy (OR=1.43, 95% CI=1.04–1.98, *P*<0.04) and anterior resection (OR=1.43, 95% CI=1.16–1.76, *P*<0.01) in rectal cancer patients. Increases in adherence to the Manual of Cancer Service Standards was associated with improved five-year survival after adjustment for the casemix factors of age, stage of disease, socioeconomic status and year of diagnosis, especially for colon cancer (HR=0.97, 95% CI=0.94–0.99 *P*<0.01). There was a similar trend of improved survival in relation to increased surgical site specialisation for rectal cancer, although the effect was not statistically significant (HR=0.93, 95% CI=0.84–1.03, *P*=0.15). In conclusion, the extent of implementation of the Calman–Hine report has been variable and its recommendations are associated with improvements in processes and outcomes of care for colorectal cancer patients.

Colorectal cancer occurs in over 34 500 people in the UK each year and it kills around 16 000 of those diagnosed (Cancer Research UK, 2005). In the mid 1990s, it became clear that the UK survival rate fell below the European average (Berrino *et al*, 1995) and that the quality of NHS care varied considerably across the country (Bull *et al*, 1997; Mella *et al*, 1997). In consequence, improving survival and ensuring a high quality of care for all has become a top priority for the Government.

The strategy by which the Government hoped to achieve this in England and Wales was outlined in the 1995 Calman–Hine report (Calman–Hine Report, 1995) and the subsequent NHS Cancer Plan (The NHS Cancer Plan, 2000). These documents recommended that care should be organised into the hands of site specialists in each relevant discipline working together in multidisciplinary cancer teams (MDTs). Detailed specifications of whom and how these teams should be composed were provided in the 1997 Improving Outcomes Guidance (IOG) in Colorectal Cancer (NHS Executive, 1997).

These reforms were unusual, as they aimed to improve outcomes through the reconfiguration of facilities and personnel, rather than through the introduction of a new health technology. The recommendations originated from data suggesting that high workload (Schrag *et al*, 2002) or specialist (Birbeck *et al*, 2002; McArdle and Hole, 2002; Smith *et al*, 2003) doctors offered better outcomes than their low workload or generalist counterparts. Unfortunately, however, the evidence available to substantiate this theory is not conclusive (Kee *et al*, 1999; Parry *et al*, 1999) and, as a consequence, not all within the health service subscribe to the idea (Singh *et al*, 1997).

Despite the equivocal evidence base, however, the reforms have been a flagship NHS policy and substantial resources have been invested in their implementation. The aim of this population-based longitudinal study was to quantify the extent to which the Calman–Hine recommendations of multidisciplinary team formation and surgical site specialisation in colorectal cancer had been translated into practice by 2000, in the Yorkshire region of the UK. In addition, the study sought to determine if these changes were, as the Government intended, associated with improvements in the outcome of colorectal cancer patients.

## PATIENTS AND METHODS

All patients diagnosed with colorectal cancer (ICD10 codes C18, C19 and C20) between 1995 and 2000 in the former Yorkshire Regional Health Authority were identified via the Northern and Yorkshire Cancer Registry Information Service (NYCRIS). Routinely recorded information about patients’ disease and its management was downloaded. Any patients for whom such information was missing (due to death certificate only registration or who were managed by their GP's, privately or outside the region) were excluded.

In 2000, 13 colorectal cancer teams provided cancer care within the study region. All patients, identified via NYCRIS, were allocated to a team based on their hospital of diagnosis or (before 1998) their initial hospital of attendance.

### Assessment of Calman–Hine implementation

Two methods of assessing Calman–Hine implementation were adopted. The first assessed the formation of multidisciplinary teams and the second the move from general surgeons to site-specialist management.

#### Growth of colorectal cancer teams

In 2001, the Department of Health published National Accreditation standards (Manual of Cancer Service Standards, 2001) based on the original IOG that detailed the criteria that the ‘ideal’ colorectal cancer team should adhere to. To assess the growth of the study's teams, a team score based on each unit's adherence to these criteria over time was developed. A questionnaire was devised which asked whether each criterion had been met in each year between 1995 and 2000. Each team was then asked to complete the questionnaire for the time period covered and, by determining the number (and percentage) of standards that had been met in each year, charts of the time-scale of team formation could be charted.

#### The move towards site specialisation

Another measure of implementation was the move towards site specialist surgical care. The surgeon performing the main surgical procedure of each patient was identified. The specialty of this surgeon was determined by looking up their entries in the 2001 Medical Directory (Medical Directory, 2001) or on the website specialistinfo.com (Specialistinfo.com, 2002). In both these resources, the consultant cites their own specialist interests. If no specialism could be identified, or doctors chose to define themselves as general surgeons, they were allocated to a specialism based on their annual median workload (*n*=11). A threshold was set for a colorectal specialist surgeon as one whose annual median workload exceeded 24 new cases per year based on the IOG (NHS Executive, 1997). The proportion of patients in each year and in each team receiving their main surgery from either a self-declared or high workload specialist was then calculated.

### Statistical methods

Multilevel (random effects) binary logistic regression models were used to assess how the Calman–Hine changes were associated with care outcomes. Models were developed with the cancer team as a random effect (at level 2), allowing for within-team correlation among patient outcomes. All models were developed within the MLwiN software (MLwiN version 2.02, 2005).

Main outcomes (dependant variables) were chosen based upon recommendations given in the IOG document (NHS Executive, 1997). This document stated that (1) the use of systemic therapy should be discussed with patients who possessed Dukes C or D cancers and who were fit enough to tolerate it; (2) radiotherapy should be used preoperatively for rectal cancer patients at high risk of recurrence and (3) anterior resection (where possible) should be the operation of choice in these patients. These measures of patient health care were, therefore, all selected as dependant variables in the form of binary outcomes (i.e., patients received the recommended care, coded 1, or they did not, coded 0). As the ultimate aim of Calman–Hine was to improve survival, multilevel proportional hazards (frailty) models (Gilthorpe *et al*, 2002) were used to assess survival at 5 years.

Covariates (explanatory variables) included age (per 10 year increase), gender, the Townsend material deprivation score of each patient (derived according to the enumeration district of residence at diagnosis), year of diagnosis, stage at diagnosis (Dukes A and B, C and D or unknown) and increases in either of the Calman–Hine implementation scores (per 25% increase in team score or surgical specialisation score). A Townsend score was unable to be allocated to 200 patients. To ensure these individuals were included in the model, they were allotted the mean Townsend score of the study population. All continuous covariates were centred (i.e., each case had the population mean of the variable arithmetically deducted from it) to improve estimation procedures (Gilthorpe and Cunningham, 2000).

Bivariate correlations among all covariates were examined to assess potential problems due to collinearity, as such collinearity might confound the analyses (Tu *et al*, 2001, 2005). Where strong correlations were observed between time (year of diagnosis) and the Calman–Hine implementation scores, analyses were undertaken across two time periods – 1995 to 2000 and 1996 to 1998, separately – in an attempt to limit the effect of collinearity. The narrower time period of 1996–1998 was chosen because the correlations between time and the Calman–Hine implementation scores were reduced and this period also corresponded to the period in which the main guidance documents about service reform in colorectal cancer were published, and the rate of change in practice towards the Calman–Hine recommendations should have been greatest.

## RESULTS

### Study population

In total, 12 358 patients were diagnosed with colorectal cancer in the study area between 1995 and 2000. Of these, 810 were excluded due to missing management information as they were managed extra-regionally (*n*=94), managed by their GP (*n*=25), treated as private patients (*n*=481), death certificate only registrations (*n*=174) or had missing NYCRIS information (*n*=36). This left a study population of 11 548 cases (93.4%). Characteristics of this population are given in Table 1.

### Degree of Calman–Hine implementation

#### Adherence to manual of cancer service standards

**Figure 1:**
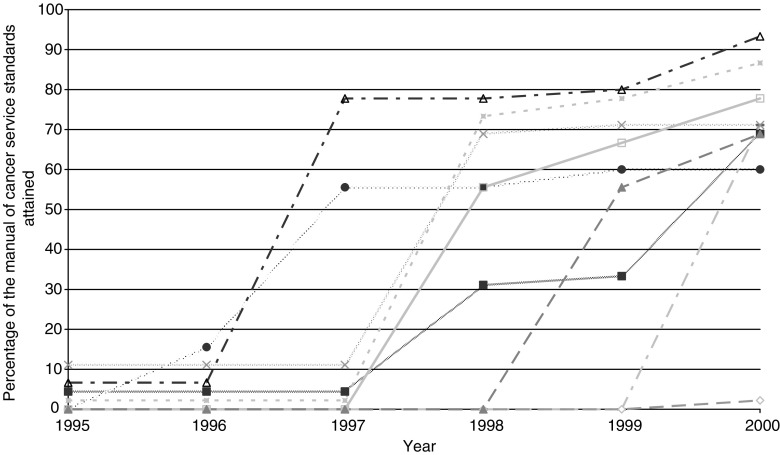
Adherence of nine Yorkshire colorectal cancer teams to the manual of cancer service standards between 1995 and 2000.

Of the 13 teams, nine completed questionnaires for the project. Figure 1 demonstrates the rate of growth of these teams. Although there was a definite change in practice over the study period, no team adhered to all the requirements of team structure and function laid out in the manual of cancer service standards by 2000.

#### Colorectal site specialisation

**Figure 2:**
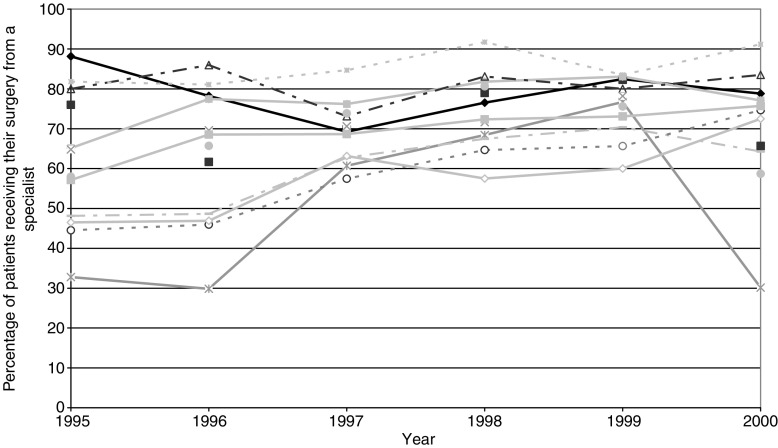
Proportion of patients in each of 13 Yorkshire colorectal cancer teams receiving their initial surgery from a colorectal specialist surgeon between 1995 and 2000.

A total of 142 surgeons performed colorectal cancer surgery on the entire study population and the overall annual median workload was seven cases per year (range 0.5–65). The annual median workload of the specialists among them was 29 (range 1 to 65). Figure 2 illustrates the proportion of patients receiving their surgery from such a specialist over the study period. Even in the final year of the study, however, a substantial proportion of patients (19%) failed to receive their initial surgery from a colorectal specialist.

### Change in process and outcomes according to extent of Calman–Hine implementation

#### Use of chemotherapy in Dukes stage C and D colorectal cancer patients

Thirty-seven per cent of the Dukes stage C and D population received some form of chemotherapy, although the rate of use varied across teams and over time (Table 2). In 1995, the median percentage of patient receiving such treatment was 34.0% (range 18.0–53.8%). By 2000, this had increased to a median of 36.2% (range 18.2–51.8%).

The results of multilevel models, summarised in Table 3, indicate what proportion of this change in practice was associated with implementation of the Calman–Hine recommendations. In models adjusting for casemix and year of diagnosis, a 25% increase in team score was associated with an 8% decrease in the odds of use of chemotherapy, although this was not statistically significant (OR=0.92, 95% CI=0.83–1.03). The correlations were, however, high in this model and so another was explored, with reduced collinearity, for the time period 1996–1998. This demonstrated a nonsignificant 16% increase in the odds of use of chemotherapy in relation to 25% increases in team score (OR=1.16, 95% CI=0.86–1.58). Collinearity had thus attenuated the association towards and beyond the null.

In contrast, a 25% increase in specialisation was associated with a significant 32% increased odds of a Dukes C/D patient receiving chemotherapy (OR=1.32, 95% CI=1.02–1.69). In the shorter duration, the trend was reduced to a nonsignificant 3% increase in the odds of administration of chemotherapy (OR=1.03, 95% CI=0.55–1.94). The impact of collinearity for this outcome, therefore, confuses the findings.

#### Preoperative radiotherapy for rectal cancer

During the full study period, 16.5% of the rectal cancer population received preoperative radiotherapy. In 1995, its median percentage use across the teams was 0.0% (range 0.0–12.5%), and by 2000 this had increased to a median of 17.1% (range 3.3–70.0%). Table 2 illustrates the change in percentage administration across the teams and the study period.

Models established to determine what proportion of the changes in pratice were associated with the Calman–Hine implementation are summarised in Table 4. An increase of 25% in the team score was associated with 58% increased odds of use of preoperative radiotherapy. However, there were again concerns over the high correlation between team score and time (year of diagnosis), which raises questions over the reliability of this assessment. Consequently, a shorter duration model (1996–1998), with substantially reduced collinearity, was explored. In this model, there was no effect of a 25% increase in team score on the use of preoperative radiotherapy (OR=0.99, 95% CI=0.75–13.2). This suggests there was no genuine association between this covariate and the outcome.

For a 25% increase in surgical specialisation, the odds of preoperative radiotherapy were elevated significantly by 43% (OR=1.43, 95% CI=1.04–1.98), suggesting that increasing surgeon specialisation is linked to an increased use of preoperative radiotherapy. The effect remained in the reduced collinearity model, covering the period 1996–1998, but was no longer statistically significant (OR=1.66, 95% CI=0.71–3.88).

#### Use of anterior resection in rectal cancer patients

In 1995, the median percentage of surgical rectal cancer patients receiving an anterior resection among all teams was 46.0% (range 30.0–60.5%), and by 2000 this had increased to a median of 53.7% (range 37.5–66.7%) (Table 2).

Results from the models developed to investigate changes in the use of anterior resection in relation to team formation and surgical site specialisation are summarised in Table 5. No relationship was observed between increasing adherence to the manual of cancer service standards and the use of anterior resection. Again, there was concern about the high correlation between the team score and year of diagnosis, so a shorter duration model with a reduced collinearity was explored. Nevertheless, the lack of an effect remained.

There was, however, a statistically significant relationship between increasing surgical site specialisation and the use of this operation. A 25% increase in the proportion of patients receiving their initial surgery from a colorectal specialist was associated with a 43% increase in the odds of use of anterior resection (OR=1.43, 95% CI=1.16–1.76). The effect remained in the reduced collinearity model (OR=1.57, 95% CI=1.00–2.49), although power was inevitably reduced and the effect was only of marginal significance.

#### Five-year survival

Across the entire study period, the overall 5-year survival rate was 38.3%. The median 5-year survival rate across teams was 38.1%, but ranged from 31.1 to 45.4%. Results for the multilevel models developed to assess whether or not survival was associated with the Calman–Hine implementations are summarised in Table 6.

A 25% increase in team score was associated with a statistically significant 3% reduction in the risk of death for all colorectal cancer patients (HR=0.97, 95% CI=0.94–0.99) and 4% for colon cancer patients (HR=0.96, 95% CI=0.93–0.99). A nonsignificant 1% reduction was observed for rectal cancer patients (HR=0.99, 95% CI=0.96–1.01).

There was a trend towards reduced risk of death in relation to increased surgical site specialisation, particularly for rectal cancer (HR=0.93, 95% CI=0.84–1.03), but the effect failed to reach statistical significance.

## DISCUSSION

### Main findings

Between 1995 and 2000, the Calman–Hine recommendations in terms of team formation and surgical site specialisation were implemented at varying rates by the colorectal cancer teams of Yorkshire. Although in some hospitals teams were functioning according to some Calman–Hine principles from the outset, in no areas were all the recommendations fully realised by the end of the study period.

Attempting to determine if these shifts in the organisation of care were associated with improvements in cancer treatment and outcome was statistically complex, due to the risk of collinearity among crucial explanatory variables. However, there was evidence to suggest that the move towards surgical site specialisation was associated with the greater use of preoperative radiotherapy and anterior resection in rectal cancer patients. Small statistically significant improvements were seen in five-year survival in relation to increasing adherence to the Manual of Cancer Service Standards, especially for colon cancer patients. There was a trend towards improved survival in relation to increasing surgical specialisation, particularly for rectal cancer patients, but the effect was not statistically significant. These changes in treatment and survival relate to 25% increases in the Calman–Hine implementation scores. The data suggest, therefore, that complete adherence to the Calman–Hine principles may improve care for colorectal cancer patients.

### Limitations of the study

Deciding on a measure that truly corresponded to the extent of implementation of the Calman–Hine ideas was difficult. Two main themes of the original report were, firstly, ensuring that site specialist doctors and nurses, rather than generalists, managed patients and, secondly, that all disciplines should meet regularly to discuss and plan the optimal care pathway for each patient. The two markers chosen were, therefore, surgical site specialisation and the extent of multidisciplinary team formation. These measures were seen to be associated with changes in different processes and outcomes of care. Surgical specialisation was linked to changes in practice, while teams were associated with an improvement in survival. This may be a consequence of the bluntness of these Calman–Hine implementation surrogates but developing sharper measures to quantify organisational change is extremely difficult. Our results were, perhaps, predictable. Surgical site specialisation is associated with greater experience and training in relation to colorectal cancer and this may result in heightened awareness of current best practice, thereby encouraging appropriate referral and collaboration with oncologists explaining the greater use of chemotherapy and radiotherapy, as well as the use of the ‘gold standard’ surgical procedures. Likewise, the measurement of the ultimate outcome – survival – was linked to the team score surrogate. No health professional works in isolation and survival is dependent on the overall package of care. Such ideal treatment is reliant not only on good surgeons or oncologists but also good anaesthetics, pathology, nursing and generally a good team. Another mechanism via which good teams may influence outcomes may be due to their influence in situations where not all team members are specialists. The weekly team meetings provide a forum for the discussion of each patient and the advice given ensures that patients are managed optimally by the nonspecialist team members too. Perhaps these reasons account for why our team score measure related to improved survival.

However, the validity of these team scores could be questioned. They were generated by the collation of data recalled and researched by each cancer team. While some were meticulous in their collection, others were (frequently due to time constraints) more haphazard and, hence, the quality of the results may have been affected. In addition, we demanded no evidence that the criteria the teams reported they adhered to had been, in fact, complied with and, in the current climate, some may have been tempted to exaggerate their adherence to the new cancer service recommendations.

There are also questions around what the team score actually measured. Its composition was based around adherence to administrative criteria laid out in the Manual of Cancer Service Standards (Manual of Cancer Service Standards, 2001). Thus, it is possible that while a team may attain all the criteria, they may still not have practised as an effective team in the way Calman–Hine envisaged. Conversely, a team could be working in a collaborative fashion but fail to adhere to any of the administrative recommendations and so have attained a poor team score. As such, the use of this team score may be rather a blunt measure.

The alternative Calman–Hine implementation score looking at surgical site specialisation may also be problematic. The definition of specialisation chosen was that the surgeons themselves declared or, if this could not be obtained, workload. The median workload of the specialist surgeons was only 29 cases per year and this could be considered low for true colorectal specialists. However, this figure may not be truly representative as some surgeons worked at the boundaries of the region and so managed patients who lived out with the NYCRIS cancer registry region. The data held by the registry may, therefore, only represent a fraction of their true patient volume and could reduce the apparent median workload. It was for this reason that a composite of self-declared specialisation and workload were used to define the measure. It was not, however, a perfect method for distinguishing between specialists and generalists and could again be a rather blunt surrogate. In the future, perhaps comparing outcomes between named team and other surgeons would be a more reliable method for distinguishing between specialists and nonspecialists.

Other issues around data quality are also present. For example, the NYCRIS data set only contained limited stage information and no data regarding patient's existing comorbidities. This could be considered a flaw as it has been shown that population-based studies that fail to make adequate adjustment for casemix often produce inflated outcomes (Halm *et al*, 2002). However, 86% of the population could be allocated to a stage group and the study population exceeded 11 500 cases. Although the possibility of bias cannot be excluded entirely and, as we have no reason to believe that there are systematic differences in the extent of disease at diagnosis across the region, the high numbers involved ensure that the results cannot be attributed to differences in casemix alone.

Similarly, we were limited by the amount of treatment information available. Again, NYCRIS collects only basic treatment details and this limited our ability to use pertinent and revealing outcome measures, for example, whether preoperative radiotherapy was administered at the optimal dosage to high-risk patients instead of whether it was given at all. This was unavoidable, even though NYCRIS possesses one of the most extensive registry treatment data sets in the UK. The lack of routine, population-based national data on cancer treatment remains a general problem.

A final problem with the study is the difficulty of distinguishing between changes in practice due to the Calman–Hine changes and those arising from other sources. For example, over the study period, several papers were published supporting the use of preoperative radiotherapy and this may account for the increased use of the treatment over time. It would be impossible to discount this effect entirely but in all the models a time factor was included in an attempt to distinguish between innate changes over time and those due to the Calman–Hine. This time factor was included in all the models and yet the Calman–Hine implementation scores remained statistically significant in many analyses. This suggests the Calman–Hine changes may have induced change over and above that occurring naturally due to changes in the medical evidence base or other organisational factors in the NHS.

### Comparison with other studies

This study provides some of the first formal evidence to demonstrate that the Calman–Hine reports recommendations have been implemented and that these changes have improved NHS cancer care. It supports previously reported work that suggests that although initiated, MDTs were still not adhering to all Calman–Hine recommendations by 2001 (Commission of Health Improvement and the Audit Commission, 2001; National Confidential Enquiry into Peri-operative Deaths, 2001; Kelly *et al*, 2003). Similarly, it supports others measuring the extent of implementation in terms of surgical site specialisation which have shown that there has been a move towards surgical specialisation but the shift is neither uniform, nor complete (Commission of Health Improvement and the Audit Commission, 2001; National Confidential Enquiry into Peri-operative Deaths, 2001; Jolly *et al*, 2001).

A strong advantage of this study over others is its use of population-based data. The majority of studies that have attempted to assess Calman–Hine reform are all centred on case series from single units or centres (Shankar *et al*, 2001; Duxbury *et al*, 2003) or surveys of selected populations (Commission of Health Improvement and the Audit Commission, 2001; National Confidential Enquiry into Peri-operative Deaths, 2001). Our study covers the changing practice in many units and centres and includes over 11 000 patients drawn from an area representative of the UK. As such, it far exceeds the numbers included in any similar work and could, therefore, be expected to provide more reliable results.

### Implication of findings

This study provides some evidence to suggest that the Calman–Hine report's recommendations have improved outcomes in colorectal cancer. However, the work also suggests that unacceptable variations in patterns of treatment and outcome remain and this must be recognised and addressed if Calman–Hine is to achieve its ultimate aim of a uniformly high standard of care for all. As there is little reason to suspect that the situation in Yorkshire is radically different to that across the rest of the England, it seems fair to assume that our results reflect the national situation. This work, therefore, provides cautious support for the current NHS policy of cancer service reorganisation.

## Figures and Tables

**Table 1:** Characteristics of the study population

	**1995–2000**		**1996–1998**	
**Characteristic**	***n***	**%**	***n***	**%**
*Tumour site*				
Colon	6879	59.6	3497	59.5
Rectosigmoid	1186	10.3	616	10.5
Rectum	3483	30.1	1763	30.0

*Gender*				
Male	6246	54.1	3147	53.6
Female	5302	45.9	2729	46.4
				
*Age (years)*				
⩽60	1979	17.1	976	16.6
61–70	2912	25.2	1496	25.5
71–80	4147	36.1	2124	36.1
⩾81	2483	21.5	1280	21.8

*Townsend quintile*^a^ *(most affluent ↓ most deprived)*				
1	2273	19.7	1157	19.7
2	2265	19.6	1156	19.7
3	2271	19.7	1157	19.7
4	2270	19.7	1157	19.7
5	2268	19.6	1155	19.7
Unknown	201	1.7	94	1.6

*Duke's stage* b				
A/B	4554	39.4	2265	38.6
C/D	5408	46.8	2741	46.7
Unknown	1546	13.7	870	14.8

a Patients’ with unknown Townsend scores were still included in the models by allocating them the mean score of the study population.

b Duke's stage was included as a categorical variable in the models using the groupings A/B, C/D or unknown.

**Table 2:** Median percentage uses of different treatments across the colorectal cancer teams

	**Chemotherapy in Dukes C and D patients**		**Preoperative radiotherapy in rectal cancer patients**		**Anterior resection in rectal cancer patients**	
**Year**	**Median**	**Range**	**Median**	**Range**	**Median**	**Range**
1995	34.0	18.0–53.8	0.0	0.0–12.5	46.0	30.0–60.5
1996	38.5	21.9–56.4	9.1	0.0–35.7	50.0	22.7–80.6
1997	35.5	14.3–50.6	11.1	0.0–50.0	59.7	25.8–78.6
1998	43.2	20.8–51.3	19.8	5.0–61.9	55.0	37.9–66.7
1999	41.4	22.2–63.6	22.7	4.0–75.0	55.8	27.3–68.8
2000	36.2	18.2–51.8	17.1	3.3–70.0	53.7	37.5–66.7

**Table 3:** Odds ratios for the use of chemotherapy in Dukes stage C and D colorectal cancer patients, in relation to the year of diagnosis and the Calman–Hine implementation scores

	**1995–2000**			**1996–1998**		
**Covariate**	**Odds ratio**	**95% Confidence interval**	***P*-value**	**Odds ratio**	**95% Confidence interval**	***P*-value**
Year of diagnosis^a^	1.18	1.14–1.22	<0.01	1.09	0.78–1.52	0.60
Team score^b^ per 25% increase	0.92	0.83–1.03	0.15	1.16	0.86–1.58	0.33
Specialisation score^b^ per 25% increase	1.32	1.02–1.69	0.03	1.03	0.55–1.94	0.92

a Odds ratios adjusted for gender, age, stage of disease, Townsend deprivation score.

b Odds ratios adjusted for gender, age, stage of disease, Townsend deprivation score and year of diagnosis.

**Table 4:** Odds ratios for the use of preoperative radiotherapy in the treatment of surgical-treated rectal cancer patients in relation to the year of diagnosis and the Calman–Hine implementation scores

	**1995–2000**			**1996–1998**		
**Covariate**	**Odds ratio**	**95% Confidence interval**	***P*-value**	**Odds ratio**	**95% Confidence interval**	***P*-value**
Year of diagnosis^a^	1.43	1.33–1.55	<0.01	1.73	1.41–2.12	<0.01
Team score^b^ per 25% increase	1.58	1.41–1.76	<0.01	0.99	0.70–1.32	0.96
Specialisation score^b^ per 25% increase	1.43	1.04–1.98	0.04	1.66	0.71–3.88	0.24

a Odds ratios adjusted for gender, age, stage of disease, Townsend deprivation score.

b Odds ratios adjusted for gender, age, stage of disease, Townsend deprivation score and year of diagnosis.

**Table 5:** Odds ratios for the use of anterior resection in surgical rectal cancer patients in relation to the year of diagnosis and the Calman–Hine implementation scores

	**1995–2000**			**1996–1998**		
**Covariate**	**Odds ratio**	**95% Confidence interval**	***P*-value**	**Odds ratio**	**95% Confidence interval**	***P*-value**
Year of diagnosis^a^	1.01	0.96–1.06	0.73	0.96	0.83–1.10	0.53
Team score^b^ per 25% increase	1.00	0.91–1.10	1.00	0.91	0.76–1.09	0.32
Specialisation score^b^ per 25% increase	1.43	1.16–1.76	<0.01	1.57	1.00–2.49	0.05

a Odds ratios adjusted for gender, age, stage of disease, Townsend deprivation score.

b Odds ratios adjusted for gender, age, stage of disease, Townsend deprivation score and year of diagnosis.

**Table 6:** Cox proportional hazards models assessing five-year survival in colorectal cancer patients diagnosed between 1995 and 2000

**Covariate**	**Hazard ratio**	**95% Confidence interval**	***P*-value**
*Colorectal cancer*			
Year of diagnosis^a^	0.98	0.97–0.99	<0.01
Team score^b^ per 25% increase	0.97	0.94–0.99	0.01
Specialisation score^b^ per 25% increase	0.98	0.93–1.04	0.54
			
*Colon cancer*			
Year of diagnosis^a^	0.98	0.96–1.00	0.02
Team score^b^ per 25% increase	0.96	0.93–0.99	<0.01
Specialisation score^b^	1.01	0.94–1.08	0.85
			
*Rectal cancer*			
Year of diagnosis^a^	0.99	0.96–1.01	0.26
Team score^b^ per 25% increase	0.99	0.95–1.04	0.81
Specialisation score^b^ per 25% increase	0.93	0.84–1.03	0.15

a Hazard ratios adjusted for gender, age, stage of disease, Townsend deprivation score.

b Hazard ratios adjusted for gender, age, stage of disease, Townsend deprivation score and year of diagnosis.
